# Ketamine rapidly relieves acute suicidal ideation in cancer patients: a randomized controlled clinical trial

**DOI:** 10.18632/oncotarget.13743

**Published:** 2016-12-01

**Authors:** Wei Fan, HaiKou Yang, Yong Sun, Jun Zhang, Guangming Li, Ying Zheng, Yi Liu

**Affiliations:** ^1^ Department of Anesthesiology, Huai’an First People's Hospital, Nanjing Medical University, Huai’an City, Jiangsu, 223300, P.R. China; ^2^ Department of Anesthesiology, Maternal and Child Health Care Hospital of Huai’an City, Qingpu, Huai’an City, Jiangsu, 223002, P.R. China; ^3^ Department of Burn and Plastic Surgery, Huai’an First People's Hospital, Nanjing Medical University, Huai’an City, Jiangsu, 223300, P.R. China

**Keywords:** ketamine, cancer, depression, suicidal ideation

## Abstract

This study was designed to examine the rapid antidepressant effects of single dose ketamine on suicidal ideation and overall depression level in patients with newly-diagnosed cancer. Forty-two patients were enrolled into the controlled trial and randomized into two groups: ketamine group and midazolam group. Patients from the two groups received a sub-anesthetic dose of racemic ketamine hydrochloride or midazolam. Suicidal ideation score, measured with the Beck Scale and suicidal part of the Montgomery-Asberg Depression Rating Scale, significantly decreased on day 1 and day 3 in ketamine-treated patients when compared to those treated with midazolam. Consistently, overall depression levels measured using the Montgomery-Asberg Depression Rating Scale indicated a significant relief of overall depression on day 1 in ketamine-treated patients. Collectively, this study provides novel information about the rapid antidepressant effect of ketamine on acute depression and suicidal ideation in newly-diagnosed cancer patients.

## INTRODUCTION

Cancer patients experience increased risk and incidence of suicide and other psychiatric disorders [1, 2]. Epidemiological studies indicate an almost doubled incidence of suicide in patients with different site-specific cancers when compared to the general population [2–7], especially within the first few months of diagnosis [7]. Immediate remission from the ideation of suicide will help to prevent unexpected, emergent suicide. However, most of the currently available antidepressants normally take weeks, if not months, to show antidepressant effects [8].

Ketamine, a general anesthetic, has recently been shown to induce a rapid onset and transient antidepressant effect in different kinds of psychiatric disorders [9–19]. The rapid-acting nature of ketamine and the solid practical validation of high rates of efficacy in treating depression lead us to hypothesize that administration of a single dose ketamine might be a potential strategy to treat acutely-developed depression and suicidal ideation in patients with newly-diagnosed cancer.

## RESULTS

### Participants basic characteristics

**Figure 1:**
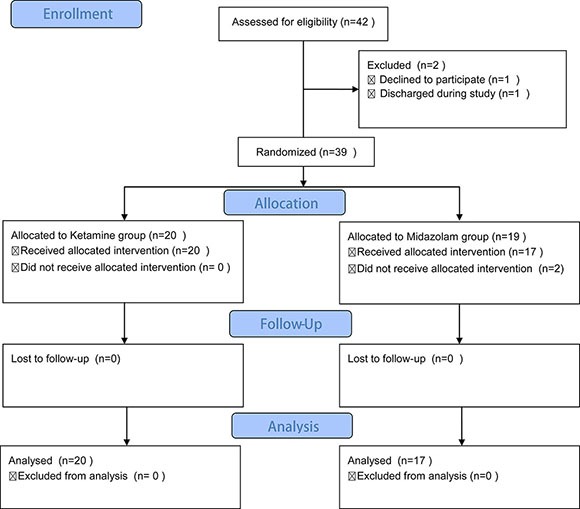
Flow diagram of the present study

**Figure 2:**
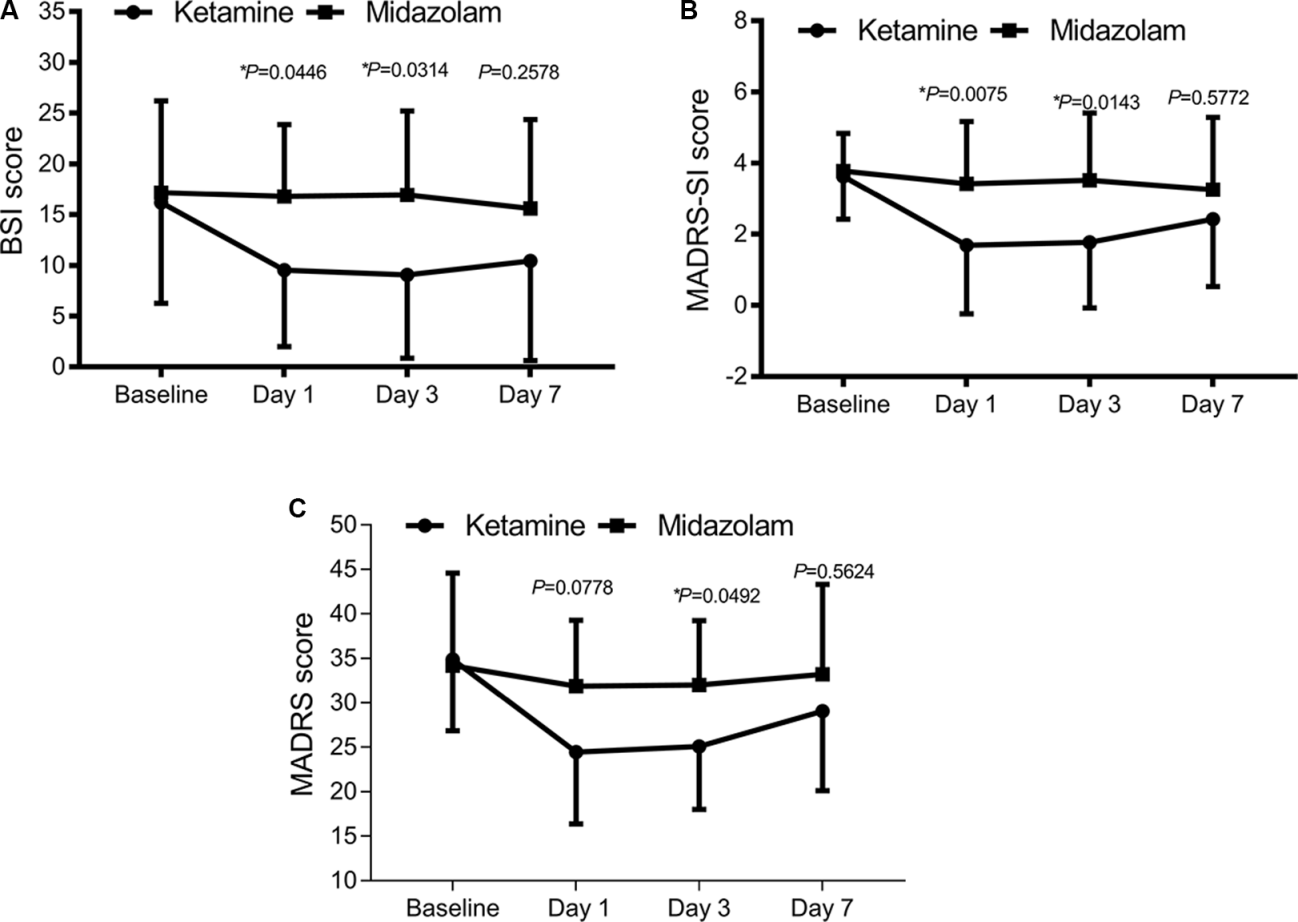
Change in suicidal ideation severity and overall depression severity following single ketamine or midazolam treatment (**A**) Suicidal ideation severity evaluated with BSI score, *P < 0.05. (**B**) Suicidal ideation severity evaluated with MADRS-SI score, *P < 0.05. (**C**) Overall depression severity evaluated with MADRS score, *P < 0.05 between the two groups.

Forty-two patients were enrolled into a controlled trial and randomized with computer-generated random numbers into two groups receiving either a sub-anesthetic dose of *i.v*. 0.5 mg/kg racemic ketamine hydrochloride or 0.05 mg/kg midazolam over 40 min using a double-blind infusion pump (Figure 1). Baseline demographic and clinical characteristics of the participants are comparable and summarized in Table 1. Baseline depression severity and suicidal ideation were in the moderate to severe range and were not different between the two treatment groups (Figure 2).

**Table 1:** Characteristic of study sample

Characteristic	Total sample (*n* = 37)	Ketamine (*n* = 20)	Midazolam (*n* = 17)	*P*-value
Age (year)	45.78 ± 14.37	46.75 ± 14.04	44.65 ± 15.1	0.6636
Female	25 (67.57 %)	12 (32.43%)	13 (35.14%)	0.3193
BSI score (baseline)	16.81 ± 1.984	17.06 ± 1.819	16.6 ± 2.137	0.4910
MADRS-SI score( baseline)	3.811 ± 1.221	3.65 ± 1.173	3.65 ± 1.268	0.3925
MADRS score (baseline)	34.66 ± 9.54	34.89 ± 8.04	34.19 ± 10.83	0.9985
Primary diagnosis				-
Lung cancer	7 (18.92%)	4 (10.81%)	3 (8.11%)	-
Gastric cancer	12 (32.43%)	7 (18.92%)	5 (13.81%)	-
Bone cancer	7 (18.92%)	5 (13.51%)	2 (5.41%)	-
Pancreas cancer	11 (29.73%)	4 (10.81%)	7 (16.92%)	-

BSI, Beck Scale for suicidal Ideation; MADRS, Montgomery-Asberg Depression Rating Scale; Values indicate mean S.D. or count (%). Variables are compared between treatment groups using unpaired student t test or *X^2^* test.

### Ketamine reduces acute suicidal ideation and overall depression

BSI (the Beck Scale for suicidal ideation) and MADRS-SI (the suicidal part of the Montgomery-Asberg Depression Rating Scale) scores were both significantly lower in the ketamine group than those in the midazolam group on day 1 (BSI: 9.53 ± 9.53 *v.s*. 16.79 ± 7.07, *P* = 0.0474; MADRS-SI: 1.69 ± 1.93 *v.s*. 3.42 ± 1.75, *P* = 0.0119) and day 3 (BSI: 9.07 ± 8.21 *v.s*. 16.93 ± 8.27, *P* = 0.0265; MADRS-SI: 1.77 ± 1.84 *v.s*. 3.52 ± 1.89, *P* = 0.0107) time points following administration (Figure 2A and 2B).

To evaluate the antidepressant effect of ketamine, MADRS score was measured. Consistently, a significant antidepressant effect of ketamine on MADRS score emerged 1 day following administration (24.46 ± 8.04 *v.s*. 31.89 ± 7.39, *P* = 0.0339) and a promising, continued antidepressant effect on day 3 (25.09 ± 7.07 *v.s*. 32.03 ± 7.21, *P* = 0.0546) following treatment. This effect was no longer significant at 7 days following treatment (Figure 2C).

## DISCUSSION

Ketamine, a high-affinity, noncompetitive *N*-methyl-d-aspartate (NMDA) receptor antagonist, has attracted widespread attention as a potential rapid-acting antidepressant. There is also considerable interest in its use for the rapid treatment of patients deemed at risk for suicide [10, 20]. In the past 10 years, evidence has emerged showing that sub-anesthetic doses of ketamine (0.5 mg/kg) induce fast-acting antidepressant effects on depressed patients [20]. Antidepressant effects of ketamine were observed as soon as 40 min after infusion and typically lasted at most for 7 days, with some patients experiencing more prolonged mood improvement [10].

In the present study, we describe acute antidepressant and anti-suicidal effects of single administration of ketamine, which were not observed in midazolam-treated group, in newly-diagnosed cancer patients during inpatient rehabilitation. Antidepressant and anti-suicidal effects of ketamine were significantly seen as soon as 1 day following administration and typically lasted for at least 3 days. We did not detect significant increases of treatment-related emergent psychiatric symptoms in patients that received ketamine during the 7 days of follow-up observations. These findings indicate that ketamine is safe and effective for short term use at a sub-anesthetic dose of 0.5 mg/kg over 40 minutes. Despite the availability of numerous monoaminergic-based antidepressants, most patients require several weeks, if not months, to respond to these treatments, and half of patients never attain sustained remission of their symptoms [8]. Considering that there are no available pharmacological agents having a similar time scale to that for ketamine, saline and midazolam are frequently-used as control agents in studying rapid-acting antidepressant effect [10]. Midazolam is a medication used for anesthesia, procedural sedation without antidepressant effect, which makes it a strong blind placebo control, so it was used as the control treatment in the present study.

The biological mechanisms underlying the rapid-acting antidepressant activity of ketamine remain largely unknown. Preclinical evidence indicates that mammalian target of rapamycin signaling [21], protein synthesis through eukaryotic translation elongation factor 2 dephosphorylation [22], as well as brain-derived neurotrophic factor increases [22, 23], underlie the rapid antidepressant responses of ketamine. Most recent evidence finds that ketamine, as an NMDA antagonist, was unexpectedly reported to exert antidepressant actions involving activation of α-amino-3-hydroxy-5-methyl-4-isoxazole propionic acid receptors, but not in a NMDARs-related manner [8]. Metabolism of ketamine to (2S,6S;2R,6R)-hydroxynorketamine is essential for its antidepressant effects [8].

Lack of long-term observations of ketamine's effect on depressive symptoms might be a limitation of our study. While chronic treatment with ketamine has been reported to prolong the antidepressant response [24–28], considering potential risk of addiction, we did not continue to administer ketamine repeatedly. In few clinical trails, ketamine-treated patients experienced transient hemodynamic effects [29–33], which indicates strict patient monitoring is needed in future studies.

## MATERIALS AND METHODS

### Participants

The trail sample was drawn from the Huai’an First People's Hospital (Huai’an, Jiangsu province) and Maternal & Child Health Care Hospital of Huai’an City (Huai’an Jiangsu province) between February 2011 and May 2016. The study was conducted in accordance with the approved guidelines from the Institutional Medical Ethics Committee of the two institutes. Written informed consent was obtained from all subjects prior to participation. Patients matching the following criteria were included in this study: between 18 and 70 years old; first diagnosed as cancer within 3 months; and basic communication capability to complete the interview. Patients were excluded if they had cardiorespiratory diseases; drug addiction history or sedative–hypnotic drug(s) use; neuropsychiatric or cognitive diseases or a related treatment history; suicidal attempts or ideation before cancer diagnosis; and family history of psychiatric history. Forty-two patients were enrolled into the controlled trial and randomized with computer-generated random numbers into two groups receiving a sub-anesthetic dose of racemic ketamine hydrochloride (041107, Jiangsu Hengrui Medicine Co.,Ltd., Lianyungang City, Jiangsu Province, P.R. China) or midazolam (H20031071, Jiangsu Enhua Pharmaceutical Industry Co., Ltd., Xuzhou City, Jiangsu Province, P. R. China.). Suicidal ideation was measured with the Beck Scale for Suicidal Ideation (BSI) score and suicidal section of the Montgomery-Asberg Depression Rating Scale (MADRS-SI). Overall depression severity was evaluated with the MADRS.

### Statistics

Data was expressed as mean ± standard deviation or percentage of the whole sample. Baseline demographic and clinical characteristics of participants were analyzed using descriptive statistics and *unpaired student's t* test or *X^2^* test as appropriate. Overall depression level and suicidal ideation evaluation at different time points were analyzed with two-way ANOVA. All statistical tests were two-sided with an alpha set at 0.05. All analyses were performed with Graph PAD Prism 7.0.

## Abbreviations

BSI: the Beck Scale for suicidal ideation
MADRS-SI: the suicidal part of the Montgomery-Asberg Depression Rating Scale
SI: suicidal ideation
NMDA: noncompetitive *N*-methyl-d-aspartate
